# Genomic characterization of the European sea bass *Dicentrarchus labrax* reveals the presence of a novel uncoupling protein (UCP) gene family member in the teleost fish lineage

**DOI:** 10.1186/1471-2148-12-62

**Published:** 2012-05-11

**Authors:** Mbaye Tine, Heiner Kuhl, Martin Jastroch, Richard Reinhardt

**Affiliations:** 1 Max Planck Institute for Molecular Genetics, Ihnestresse 63-73, 14195, Berlin, Germany; 2 Helmholtz Zentrum Munich, German Research Center for Environmental Health (GmbH) Ingolstädter, Landstr. 1, Munich, Germany; 3 Genome Centre Cologne at MPI for Plant Breeding Research, Carl-von-Linné-Weg 10, 50829, Cologne, Germany

## Abstract

**Background:**

Uncoupling proteins (UCP) are evolutionary conserved mitochondrial carriers that control energy metabolism and therefore play important roles in several physiological processes such as thermogenesis, regulation of reactive oxygen species (ROS), growth control, lipid metabolism and regulation of insulin secretion. Despite their importance in various physiological processes, their molecular function remains controversial. The evolution and phylogenetic distribution may assist to identify their general biological function and structure-function relationships. The exact number of uncoupling protein genes in the fish genome and their evolution is unresolved.

**Results:**

Here we report the first characterisation of UCP gene family members in sea bass, *Dicentrarchus labrax*, and then retrace the evolution of the protein family in vertebrates. Four UCP genes that are shared by five other fish species were identified in sea bass genome. Phylogenetic reconstitution among vertebrate species and synteny analysis revealed that UCP1, UCP2 and UCP3 evolved from duplication events that occurred in the common ancestor of vertebrates, whereas the novel fourth UCP originated specifically in the teleost lineage. Functional divergence analysis among teleost species revealed specific amino acid positions that have been subjected to altered functional constraints after duplications.

**Conclusions:**

This work provides the first unambiguous evidence for the presence of a fourth UCP gene in teleost fish genome and brings new insights into the evolutionary history of the gene family. Our results suggest functional divergence among paralogues which might result from long-term and differential selective pressures, and therefore, provide the indication that UCP genes may have diverse physiological functions in teleost fishes. Further experimental analysis of the critical amino acids identified here may provide valuable information on the physiological functions of UCP genes.

## Background

Gene duplication via single gene, larger-scale fragment or whole genome duplication is known as a major evolutionary force for creating new genes [1-3]. While the “parental” copy conserves its original function, duplicated genes may undergo functional diversification by evolving new functions and therefore offer the organisms the ability to adapt to new environmental conditions [4-8]. Duplicated genes are hierarchically gathered in gene families in the genome whose expansion will depend on the number of duplication rounds and the force of selection pressures acting on duplicates resulting in their retention or loss [9]. Unconstrained duplicates may accumulate degenerative mutations and will be therefore eliminated from the genome by purifying selection [10,11].

Uncoupling proteins (UCP) are evolutionary conserved proteins involved in the coupling mechanism of the electron transport chain to ATP synthesis which is driven by a proton gradient over the mitochondrial inner membrane. The energy harvested from electrons generates a proton gradient that is used to produce ATP or, by influence of UCPs, may be dissipated as heat [12]. Heat production by mammalian UCP1 is crucial for non-shivering thermogenesis and cold-survival of rodents, hibernators and human infants [13] while the physiological role of the UCP1 orthologue in ectotherms is unclear.

Several UCP1 homologues including UCP2 and UCP3 have been identified from various tissues in vertebrates. UCP2 with a ubiquitous expression pattern has been implicated in multiple functions including the regulation of reactive oxygen species (ROS) [14,15], growth control [16,17], lipid metabolism [18,19] and regulation of insulin secretion [20]. UCP3, adjacent to UCP2 in genomes and mainly expressed in the skeletal muscle, is related to oxidative and metabolic processes [21].

In fishes, three members of the UCP gene family (UCP1, UCP2 and UCP3) have been unequivocally identified in many species including zebrafish (*Danio rerio*), the puffer fish (*Takifugu rubripes*), the rainbow trout (*Oncorhynchus mykiss*) and the gilthead sea bream (*Sparus aurata*) [22-26]. Recent studies have indicated that zebrafish genome contains more than three UCPs. Hughes and Criscuolo [27] reported the presence of a fourth gene encoding a UCP protein (UCP4). The same authors further showed that the fourth zebrafish is flanked by the same genes like other vertebrate UCP1, which implies that this latter might be an isoform of UCP1 that resulted from alternative splicing or wrong annotation. Interestingly, the sequence of the fourth zebrafish UCP (present in GeneBank) corresponds exactly to one of the two isoforms predicted as UCP4 in Ensembl genome browser, which has been previously identified as UCP1 orthologue in fish by Jastroch et al. [25]. More recently, Tseng et al. [28] indicated the presence of a fourth UCP (UCP4: different from that previously reported by Hughes and Criscuolo [27]) and a fifth UCP gene (UCP5) in zebrafish. While their phylogenetic analyses showed that zebrafish UCP4 and UCP5 are grouped in the same clades than their mammal counterparts, the authors found that these two carriers are distantly related to UCP1-UCP3 and the syntenies around are not conserved across mammals, birds, amphibians and fishes, even within teleost lineage. This indicates that names given in the literature to these genes as members of UCP family could be misleading. Hence, the exact number of uncoupling protein genes in fish genome and the evolutionary history of this protein family remain controversial and should be elucidated. The current availability of five sequenced teleost genomes offers the opportunity of performing a range of comparative and evolutionary analyses that would enable to clarify that question.

The accurate identification of unambiguous orthologue genes across species is a critical step for comparative studies aimed at inferring the molecular function of protein sequences and finding genes that are functionally related [29,30]. The traditional approaches used to establish orthology relationships of gene families are mainly based on constructing multiple alignments of sequences identified by reciprocal best BLAST hits between multiple genomes, which are then used to infer phylogenetic relationships via maximum likelihood or parsimony methods [31,32]. Although this revolutionary approach enables an accurate orthology assignment between genes, its reliability can be limited when dealing with complex genomes and large gene families, particularly in vertebrate where many genes are partial or imperfectly annotated due the complexity of gene organisation (coding sequences consisted of several exons alternating with intron sequences). Moreover, the BLAST hit searches often return the closest hits which may not have been evolved from a common ancestral gene [33]. A common complementation of this procedure is using synteny-based methods which exploit synteny information to resolve ambiguous orthology assignment between genes in different species [34]. It is worth noting that this approach has some limitations, especially related to large-scale genomic rearrangements and gain or loss of genes that can shuffle synteny information even between closely related species, but also to the incompleteness of many sequenced genomes that renders the detection of conserved synteny complex and difficult. Although the above mentioned methods appear to be somewhat limited, they benefit from a high degree of complementarity that makes them the most reliable and accurate approaches for orthology assignment when applied in parallel.

Our experimental research focuses on the European sea bass, *Dicentrarchus labrax*, which is a major marine aquaculture species in the European Union. Sea bass UCPs may provide potential targets to impact metabolism and growth, as studies of mammalian UCPs suggest major roles in energy balance and oxidative stress. To our knowledge, there has not been any report on the molecular characterisation of uncoupling protein genes in the sea bass. In this study, we have characterised four members of the uncoupling protein gene family in sea bass. The genomic structure of these four sea bass UCPs have been determined and their orthologues in the available teleost fish genomes have been identified by similarity and conserved synteny searches. Furthermore, we took advantage to the current availability of a large amount of sequence information in many species to re-annotate and retrace the evolution of UCPs in vertebrates. Our results revealed for the first time a fish-specific duplication of uncoupling protein genes.

## Results

### Annotation and nomenclature of sea bass UCPs

**Figure 1:**
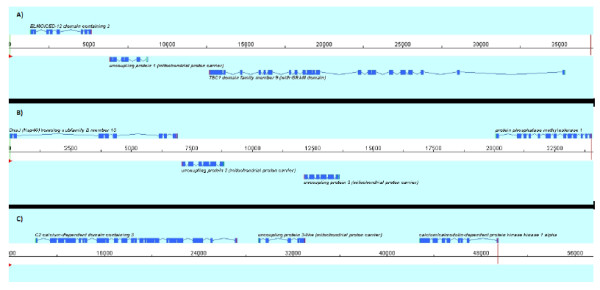
**Annotated uncoupling protein genes and their flanking genes in sea bass as displayed by Apollo Genome Annotation Curation Tool.** UCP: uncoupling protein; ELMOD2: ELMO/CED-12 domain containing 2; TBC1D9: TBC1 domain family, member 9; DNAJB13: DnaJ (Hsp40) related, subfamily B, member 13; PPME1: Protein phosphatase methylesterase 1; CAMKK1: calcium/calmodulin-dependent protein kinase kinase 1; C2CD3: C2 calcium-dependent domain containing 3.

The blast search of stickleback UCP1 against sea bass genome shotgun and BACend sequences allows the identification of three scaffolds putatively containing a UCP gene, which are respectively assigned to sea bass chromosomes LG7, LG14 and LG13. The UCP genes as well as their flanking genes were predicted using a combination of *ab initio* and homology-based predictions. UCP1 was predicted on chromosome LG7 and is flanked upstream by ELMOD2 (ELMO/CED-12 domain containing 2) and downstream by TBC1D9 (TBC1 domain family, member 9) (Figure 1A). UCP2 and UCP3 were predicted on chromosome LG14 and were found adjacent as previously demonstrated in many vertebrates including fish. They are flanked by DNAJB13 (DnaJ (Hsp40) related, subfamily B, member 13) and PPME1 (Protein phosphatase methylesterase 1) (Figure 1B). UCP3L predicted on chromosome LG13 is bounded upstream by CAMKK1 (calcium/calmodulin-dependent protein kinase kinase 1). Downstream, UCP3L is flanked by C2CD3 (C2 calcium-dependent domain containing 3) (Figure 1C). The embl files of sea bass UCP and flanking genes are available in Additional file 1.

The analysis of gene structure revealed that coding sequences of each sea bass UCP gene covered six exons as previously demonstrated in other teleost species for UCP1, UCP2 and UCP3 (Table 1). For each UCP member, Exon 2 is longer whereas Exon 4 is shorter. The comparison between four members showed that the three last exons (exons 4, 5 and 6) are conserved in terms of length. Exon 3 has the same size for UCP1, 2 and 3 but it is 6 bp longer for UCP3L. By contrast, Exon 1 is one base pair shorter for UCP3L than for UCP1, 2 and 3, which have the same size. Exon 2, the only exon different in terms of length between the four UCP members, is shorter for UCP1 and longer for UCP3L.

**Table 1:** Exon length of sea bass UCP genes

**Gene name**	**Exon1**	**Exon2**	**Exon3**	**Exon4**	**Exon5**	**Exon6**
UCP1	126	199	198	102	181	115
UCP2	126	217	198	102	181	115
UCP3	126	202	198	102	181	115
UCP3L	125	208	204	102	181	115

### Identification of UCP orthologues in other teleost species

**Figure 2:**
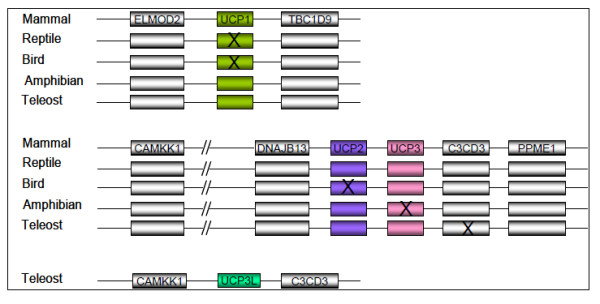
**Genomic structure encompassing UCP1, UCP2, UCP3 and UCP3L orthologues.** The UCP protein sequences were obtained from Ensembl database. ELMOD2: ELMO/CED-12 domain containing 2; TBC1D9: TBC1 domain family, member 9; DNAJB13: DnaJ (Hsp40) related, subfamily B, member 13; PPME1: Protein phosphatase methylesterase 1; CAMKK1: calcium/calmodulin-dependent protein kinase kinase 1; C2CD3: C2 calcium-dependent domain containing 3. The symbol (X) indicates genes that have been lost. Thus, UCP1 has been lost in reptile and bird lineages whereas UCP2 and UCP3 have been lost in bird and amphibian, respectively. C2CD3 (the downstream flanking gene of UCP2/UCP3 in mammal, reptile, bird and amphibian) has been lost in teleost fishes.

We have taken a closer look in other teleost genomes available in Ensembl genome browser [http://www.ensembl.org/index.html] and found that these UCP genes are located in conserved syntenic regions (Figure 2). By searching in these syntenic regions, we found the orthologues of the four UCP genes characterised in sea bass apart from UCP3L, which was not found in zebrafish. The protein sequence corresponding to UCP3L was not found for zebrafish neither in Ensembl genome browser database nor in GenBank. However, we did find the flanking genes (CAMKK1; [Protein ID: ENSDARP00000111677] and C2CD3; [Protein ID: ENSDARP00000102964]) of this fourth UCP on zebrafish chromosome 15. In order to determine whether the absence of UCP3L in zebrafish genome is due to an independent loss or a missannotation, we extracted the region harbouring CAMKK1 and C2CD3 loci and re-annotated it using GENSCAN. As for the homology searches, this did not enable prediction of another UCP gene, suggesting that UCP3L has been independently lost in the zebrafish genome. The genomic location of these UCP orthologues as well as their protein IDs are shown in Table 2. Interestingly, these UCP members are flanked in each species by the same genes as their sea bass orthologues (Figure 2).

**Table 2:** Listing and genomic location of uncoupling protein sequences used in this study

		**Genomic**	**location**			**Protein ID**		
Species	UCP1	UCP2	UCP3	UCP3L	UCP1	UCP2	UCP3	UCP3l
Takifugu	Scaffold_17	Scaffold_6	Scaffold_6	Scaffold_271	ENSTRUP00000033443	ENSTRUP00000037074	ENSTRUP00000037001	ENSTRUP00000017095
Tetraodon	Chrom 18	Chrom 7	Chrom 7	Chrom 16	ENSTNIP00000009630	ENSTNIP00000014758	ENSTNIP00000014759	ENSTNIP00000019332
Sea bass	Chrom 7	Chrom 14	Chrom 14	Chrom 13	DLA_UCP1	DLA_UCP2	DLA_UCP3	DLA_UCP3L
Stickleback	GroupIX	GroupVII	GroupVII	GroupI	ENSGACP00000022833	ENSGACP00000026903	ENSGACP00000026900	ENSGACP00000014905
Medaka	Scaffold5883	Chrom 14	Chrom 14	Chrom 13	ENSORLP00000023151	ENSORLP00000011390	ENSORLP00000011396	ENSORLP00000005419
Zebrafish	Chrom 1	Chrom 10	Chrom 10		ENSDARP00000037614	ENSDARP00000063358	ENSDARP00000105553	
Lamprey		LyEST591				CO548809.1		
Lancelet							XP_002595825	
Anole Lizard		Scaffold GL344128.1	Scaffold GL344128.1			ENSACAP00000006358	ENSACAP00000009237	
Xenopus	Scaffold_16	Scaffold_1014			ENSXETP00000032640	ENSXETP00000055772		
Checken			Chrom 1				ENSGALP00000027932	
Mouse	Chrom 8	Chrom 7	Chrom 7		ENSMUSP00000034146	ENSMUSP00000120967	ENSMUSP00000032958	
Rat	Chrom 19	Chrom 1	Chrom 1		ENSRNOP00000004900	ENSRNOP00000024156	ENSRNOP00000024005	
Cow	Chrom 17	Chrom 15	Chrom 15		ENSBTAP00000006097	ENSBTAP00000004810	ENSBTAP00000006918	
Dog	Chrom 19	Chrom 21	Chrom 21		ENSCAFP00000005489	ENSCAFP00000008312	ENSCAFP00000008310	
Elephant	S.C.s_14	S.C.s_79	S.C.s_79		ENSLAFP00000005947	ENSLAFP00000013302	ENSLAFP00000004523	
Human	Chrom 4	Chrom 11	Chrom 11		ENSP00000262999	ENSP00000312029	ENSP00000323740	

Protein IDs are given to allow access to the protein sequence on Ensembl or GenBank website.

### Sequence identity between sea bass and stickleback UCPs

**Figure 3:**
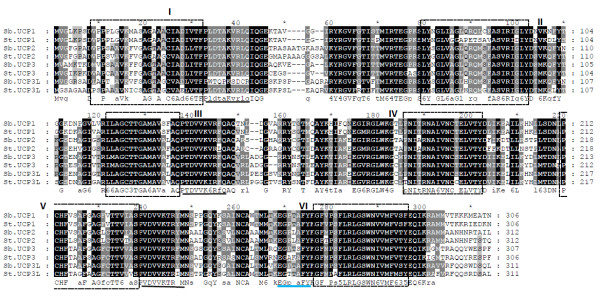
**Amino acid sequence alignment of sea bass and stickleback UCP1, UCP2, UCP3 and UCP3L.** The alignment was conducted using MAFFT version 6 and illustrated with GeneDoc. The most conserved amino acids are highlighted in black. Putative transmembrane conserved domains [23,35] are delimited with box and numbered using Roman numerals. Proton carrier signatures and the potential purine binding domain are underlined with black and blue lines, respectively.

Amino acid sequence comparison with stickleback showed that sea bass UCP genes possess all the domain amino acid residues that serve as a signature for the UCP gene core family (Figure 3). Protein sequence alignment revealed that sea bass UCP1 share more identity with stickleback UCP1 by comparison to other sea bass UCP members (Table 3). Likewise, sea bass UCP2 and UCP3 are closely related to stickleback UCP2 and UCP3, respectively compared to sea bass UCP1 and UCP3L. Similar results were observed for UCP3L which is more identical to stickleback UCP3L. The intra-specific sequence comparison in sea bass showed that UCP2 is more identical to UCP3 whereas the latter is more related to UCP3L (Table 3). Among these three UCP members, UCP2 shares more identity with UCP1. Similar results were observed in stickleback with UCP3L closer to UCP3, which is more related to UCP2 and the later more identical to UCP1 by comparison to UCP3 and UCP3L.

**Table 3:** Identity matrix between UCP genes from sea bass and stickleback

**Sequence**	**Sb.UCP1**	**Sb.UCP2**	**Sb.UCP3**	**Sb.UCP3L**	**St.UCP1**	**St.UCP2**	**St.UCP3**	**St.UCP3L**
Sb.UCP1	ID	0.692	0.684	0.630	0.718	0.669	0.667	0.620
Sb.UCP2	0.692	ID	0.711	0.643	0.557	0.891	0.701	0.675
Sb.UCP3	0.684	0.771	ID	0.720	0.557	0.705	0.872	0.720
Sb.UCP3L	0.630	0.643	0.720	ID	0.545	0.621	0.678	0.755
St.UCP1	0.718	0.557	0.557	0.545	ID	0.538	0.532	0.524
St.UCP2	0.669	0.891	0.705	0.621	0.538	ID	0.689	0.646
St.UCP3	0.667	0.701	0.872	0.678	0.532	0.689	ID	0.694
St.UCP3L	0.620	0.675	0.720	0.755	0.524	0.646	0.694	ID

ID: identical; Sb: sea bass; St: stickleback.

### Identification of teleost UCP orthologues in other vertebrates

The search for UCP orthologues in vertebrates enabled their identification in a variety of species including mammalians, reptiles, birds and amphibians. The chromosomal location of UCPs and the protein ID in each species are shown in Table 2. The search for UCP1 ortologues in vertebrates enabled its identification in mammals (Human, Mouse, etc.). In amphibians (*Xenopus tropicalis*), UCP1 was found. However, no UCP1 orthologue sequence was found in birds and reptiles and the synteny search of the region containing flanking genes in the chicken (*Gallus gallus*) and the Anole lizard (*Anolis carolinensis*) did not allow identification of sequences similar to UCP1. UCP2 and UCP3 are present in mammals (Human, Elephant, Crow, Dog, Rat and Mouse) and reptiles (Anole lizard), and are adjacent as previously demonstrated in many reports. As in teleost fishes, they are flanked upstream by the same gene (DNAJB13) but they are bounded in their downstream part by C2CD3 located just before PPME1 (Figure 2). In amphibian genomes, only UCP2 was found and it is flanked by the same genes as reptile and mammalian UCP2/UCP3. The re-annotation of the region bounded by the flanking genes by GENSCAN did not enable prediction of another UCP gene, supporting the results of homology searches. Contrary to amphibians, birds have only UCP3 which flanked by the same genes as amphibian UCP2 (Figure 2). Our re-annotation of the region surrounded by the flanking genes did not allow the prediction of new genes. Our search for teleost UCP orthologues in lamprey allows the identification of one UCP gene only, a sequence that has been characterized UCP2 by Wang et al. [36]. We then searched for UCP orthologues in the lancelet which is considered as the closest living invertebrate relative to vertebrates. This allowed the identification of a UCP-like sequence *Accession number: ABR10900* in *Branchiostoma belcheri*. ClustalW multiple alignments revealed that the lancelet UCP-like sequence was 53%, 55% and 56% identical to sea bass UCP1, UCP2 and UCP3, respectively. Comparisons with all vertebrate UCP sequences revealed that the lancelet UCP-like shares more identity (57.6%) with cow (*Bos taurus*) UCP3. However, these results are in contradiction with those from the phylogenetic analysis including the UCP-like (results not shown) because the latter indicated that lancelet UCP is closer to vertebrate UCP1.

### Phylogenetic analysis

**Figure 4:**
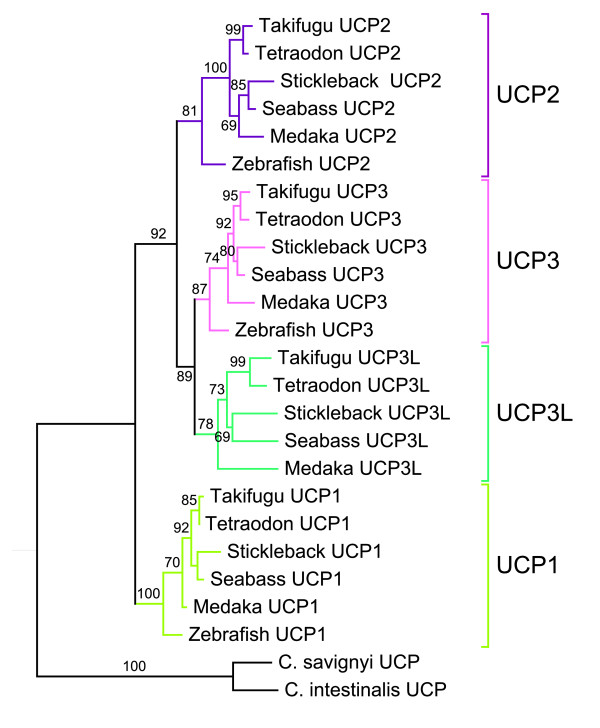
**Maximum likelihood tree of teleost UCP proteins.** The phylogenetic tree was conducted with 23 amino acid sequences using PhyML program. The values at the nodes represent bootstrap percentages from 1000 replicates. The tree was rooted with invertebrate (*Ciona intestinalis*, *Ciona savignyi* and *Caenorhabditis elegans*) UCP sequences.

**Figure 5:**
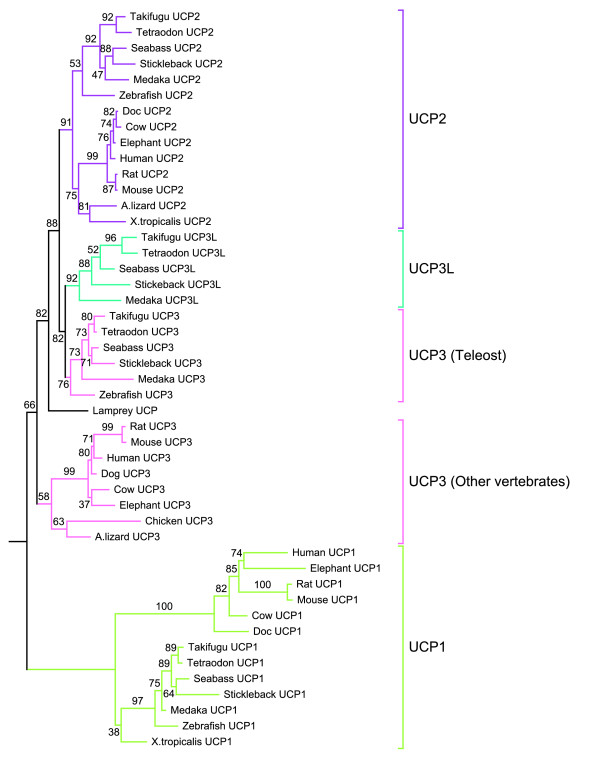
**Maximum likelihood tree depicting the phylogenetic relationship of vertebrate UCP proteins.** The phylogenetic tree was conducted with 47 amino acid sequences using PhyML program. The values at the nodes represent bootstrap percentages from 1000 replicates. The tree was rooted with midpoint rooting.

Phylogenetic trees of UCP proteins from the predicted genes in sea bass and the other five teleosts as well as sequences of vertebrates from Ensembl and GenBank were constructed using maximum likelihood (ML) method as implemented in PhyML. All fish UCPs fall into four clades that are respectively comprised of UCP1, UCP2, UCP3 and UCP3L (Figure 4). The branching patterns within these clusters matched the phylogenetic relationships among the six teleost species analysed in this study [37]. In Negrisolo et al. [37] study where zebrafish was used as an out-group, stickleback and sea bass clustered together and are closer to medaka, which is more distantly related to the tetraodontiform clade (Tetraondon and Takifugu). These four clades are consistent with the results of sequences similarity between sea bass and stickleback which showed that the same UCP members from different species share more identity than different members of the same species (Table 3). The phylogenetic analysis including other vertebrates (Figure 5) confirmed that UCP3L is newly annotated and found only in teleost fishes. The phylogenetic inference based on UCP protein sequences grouped teleost UCP1 in the same clade with their vertebrate orthologues (Figure 5). Likewise, the UCP2 orthologues from all vertebrates including teleost are assigned to the same clade. Teleost UCP3 are grouped in the same clade, which was separated with mammal UCP3 clade with a high bootstrap support. This phylogenetic pattern was confirmed by the tree inferred from nucleotide sequences (results not shown).

### Selection pressures

The ratios of nonsynonymous (d_N_) to synonymous (d_S_) substitution (ω = d_N_/d_S_) were calculated for each teleost UCP cluster. All pairwise comparisons of teleost orthologues are shown in Additional file 2. The average values of ω were 0.393, 0.469, 0.387 and 0.883 for UCP1, UCP2, UCP3 and UCP3L, respectively. According to ω values estimated from the four UCP sequences, we found that UCP3L has the highest d_N_/d_S_ ratio (0.883) whereas UCP3 has the lowest ratio (0.387). The ω value of UCP3L is about twice the magnitude of the other UCP members. All UCP genes have a d_N_/d_S_ ratio < 1 apart from UCP3L whose two pairwise comparisons (Seabass/Medaka and Takifugu/Medaka) showed d_N_/d_S_ ratios > 1 (see Additional file 2). The global d_N_/d_S_ ratio estimated using REL analysis showed similar results with higher value for UCP3L (ω = 0.890) compared to UCP1 (ω = 0.159), UCP2 (ω = 0.183) and UCP3 (ω = 0.135). The REL analysis found no positively selected sites for UCP1 and UCP3L. One site was positively selected from the REL analysis for UCP2 (codon 47) and UCP3 (codon 270) with a BF ≥ 50, but they were not confirmed as exhibiting very strong evidence for positive selection (BF ≥ 100). The REL analysis indicated that all UCP1 sites are under strong purifying section. The number of sites identified from REL analysis as being under strong purifying selection (BF ≥ 100) was 168, 112 and 71 for UCP2, UCP3 and UCP3L, respectively.

### Functional divergence

**Figure 6:**
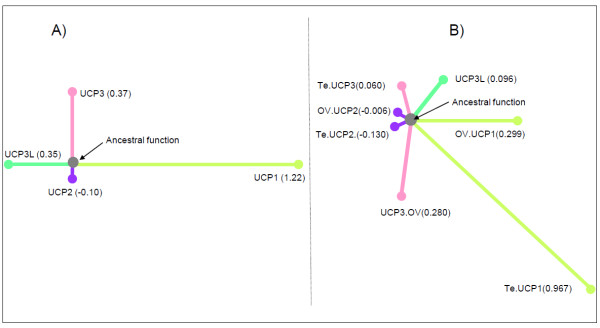
**Schematic representation of functional distance between UCP paralogues in teleost fishes (A) and in major vertebrate lineages including teleosts (B).** The functional branch length (bF) of UCP genes was estimated using DIVERGE v.2.0. Te: teleost; OV: other vertebrates.

Table 4 shows the coefficients of type-I and type-II functional divergence (θ_I_ and θ_II_) of pairwise comparisons between the members of the UCP family. All pairwise comparisons showed θ_I_ values significantly greater than zero (*P* < 0.05), which provides evidence of type-I functional divergence between UCP clusters in teleost fishes. To determine which UCP cluster is functionally more divergent, we performed functional distance (bF) analysis. The functional branch length (bF) of UCP1, UCP3, UCP3L and UCP2 is 1.22, 0.37, 0.35 and −0.10, respectively (Figure 6A). The long bF of UCP1 and UCP3 and UCP3L (to a lesser extent) indicates substantial altered functional constraints in these clusters whereas virtual zero of UCP2 bF indicated that the evolutionary rate at each site in this duplicate cluster is almost identical to the ancestral gene. The analysis including vertebrate orthologues showed similar results with a longer bF for UCP1 followed by UCP3 (Figure 6B). As for their teleost orthologue, vertebrate UCP2 showed a bF virtually equal to zero. Comparisons between groups indicated UCP1 bF was larger in teleosts whereas UCP3 bF was longer in the other vertebrates. Contrary to θ_I_, all pairwise comparisons showed extremely small values for θ_II_, indicating no clear evidence of the occurrence of type-II functional divergence between any UCP clusters in teleost fishes.

**Table 4:** **Type-I (θ**_**I**_ **± standard error, upper right diagonal) and type-II (θ**_**II**_ **± standard error, lower left diagonal) functional divergence between UCP clusters in teleost fishes**

**Gene name**	**UCP1**	**UCP2**	**UCP3**	**UCP3L**
UCP1		0.559 ± 0.126*	0.846 ± 0.123*	0.729 ± 0.126*
UCP2	0.063 ± 0.046		0.205 ± 0.096*	0.291 ± 0.109*
UCP3	0.128 ± 0.044	0.016 ± 0.045		0.242 ± 0.121*
UCP3L	0.113 ± 0.057	−0.003 ± 0.060	−0.007 ± 0.058	

θ_I_ and θ_II_ were estimated using DIVERGE program and significant values (*P* < 0.05) are labelled with *.

Moreover, critical amino acid residues that might be responsible for the functional divergence of the UCP family in teleost were predicted by calculating the site-specific profile based on posterior probability analysis. A posterior probability value higher than 0.7 (Q_I_ (k) > 0.7) was used as a cutoff to identify potential sites of type-I functional divergence in all pairwise comparisons between UCP clusters. Among 283 sites analysed in all pairwise comparisons, 235 amino acid residues (44 of which with Q_I_ (k) > 0.9) were predicted as being responsible for site-specific difference (type-I functional divergence) between UCP1/UCP3 clusters (see Additional file 3). The comparisons of UCP1 with UCP2 and UCP3L allowed the identification of 16 and 224 potential sites of type-I divergence, respectively. For the UCP2/UCP3L and UCP3/UCP3L comparisons, three potential sites that have undergone shifted rates have been identified whereas only one site is predicted to be highly functional divergence-related between UCP3 and UCP3L.

Overall, there was no clear evidence for type-II functional divergence but we did further investigate whether it exists any potential site for type-II functional divergence. We applied a cutoff of Q_II_ (k) > 1 for site-specific posterior probabilities. Thus, a total of 46 radical changes were identified between all pairwise comparisons (see Additional file 3). Amongst them, the largest number and highest posterior probability values were found in the comparisons with UCP1 (UCP1/UCP3, UCP1/UCP3L and UCP1/UCP2). Only two potential type-II sites were respectively detected in UCP2/UCP3 comparison. No radical change was found in UCP2/UCP3L and UCP3/UCP3L comparisons.

## Discussion

Five so-called UCP homologues UCP1-UCP5 annotated in mammals have been reported in the zebrafish, *Danio rerio*. While zebrafish UCP1-UCP3 are closely related, UCP4 and UCP5 do not group into the core UCP family [28]. Accordingly, Nedergaard and Cannon [38] had already suggested that these carriers (UCP4 and UCP5) are wrongly annotated as UCP and do not belong to the uncoupling protein family. In this study, we have identified four different UCP genes closely related in the European sea bass *D. labrax*. Our further analyses revealed these UCP genes are shared by five other fish species, providing evidence for the existence of four unambiguous UCP gene family members in teleost organisms. Similarity searches revealed that teleost UCP1, UCP2 and UCP3 are clearly orthologues to the corresponding UCP genes in the other vertebrates including amphibians, birds, reptiles and mammals. The detailed analysis of conserved synteny of the region harbouring UCP loci confirmed the similarity results. Mammal, reptile, bird and amphibian UCP1 were flanked with the same genes (ELMOD2, upstream and TBC1D9, downstream) like in teleost fishes. The upstream flanking gene of UCP2/UCP3 was the same (DNAJB13) for teleosts and the other vertebrates, but their downstream flanking was different. Mammal, reptile, bird and amphibian UCP2/UCP3 were bounded upstream by C2CD3 located just before PPME1 that flanks teleost UCP2/UCP3, suggesting that C2CD3 has been lost in teleost fishes. The phylogenetic analyses of vertebrate UCP genes also support the similarity results, because they have enabled the unequivocal identification of the orthologues of these three teleost UCPs in a variety of other vertebrate species. The maximum likelihood analysis revealed that teleost UCP2 was monophyletic with UCP2 of other vertebrates including amphibians and reptiles. This monophyletic character together with the high level of sequence identity within vertebrate UCP2, suggests evolutionary conserved functions. This is in good agreement with the virtual zero of its functional branch length (Figure 6A) that indicates this cluster may have maintained a larger component of ancestral function. Likewise, teleost UCP1 was monophyletic with amphibian and mammalian UCP1 as previously reported by many authors [24,27,39]. The UCP1 clade is highly divergent from that of other UCP paralogues as evidenced by its longest branch length, which is indicative of strong selective constraints and functional shifts [40]. This interpretation is in good agreement with our functional analyses that indicated large site-specific differences (type-I functional divergence) and higher posterior probability values (type-II functional divergence) in all pairwise comparisons including UCP1. The longer bF of UCP1 in vertebrates also suggests important functional shifts, which are more marked in teleosts. Mammalian, reptile and bird UCP3 are monophyletic, but their monophyly with their teleost orthologues was not resolved. These observations suggest that teleost UCP3 are highly divergent from that of the other vertebrates, divergence that may be due to an accelerated evolutionary rate of UCP3 either in teleost or in other vertebrates. The functional analyses showed a longer functional branch length in the other vertebrates compared to teleost (bF = 0.280 vs. 0. 060; Figure 6B), suggesting a functional shift during the evolution of UCP3.

**Figure 7:**
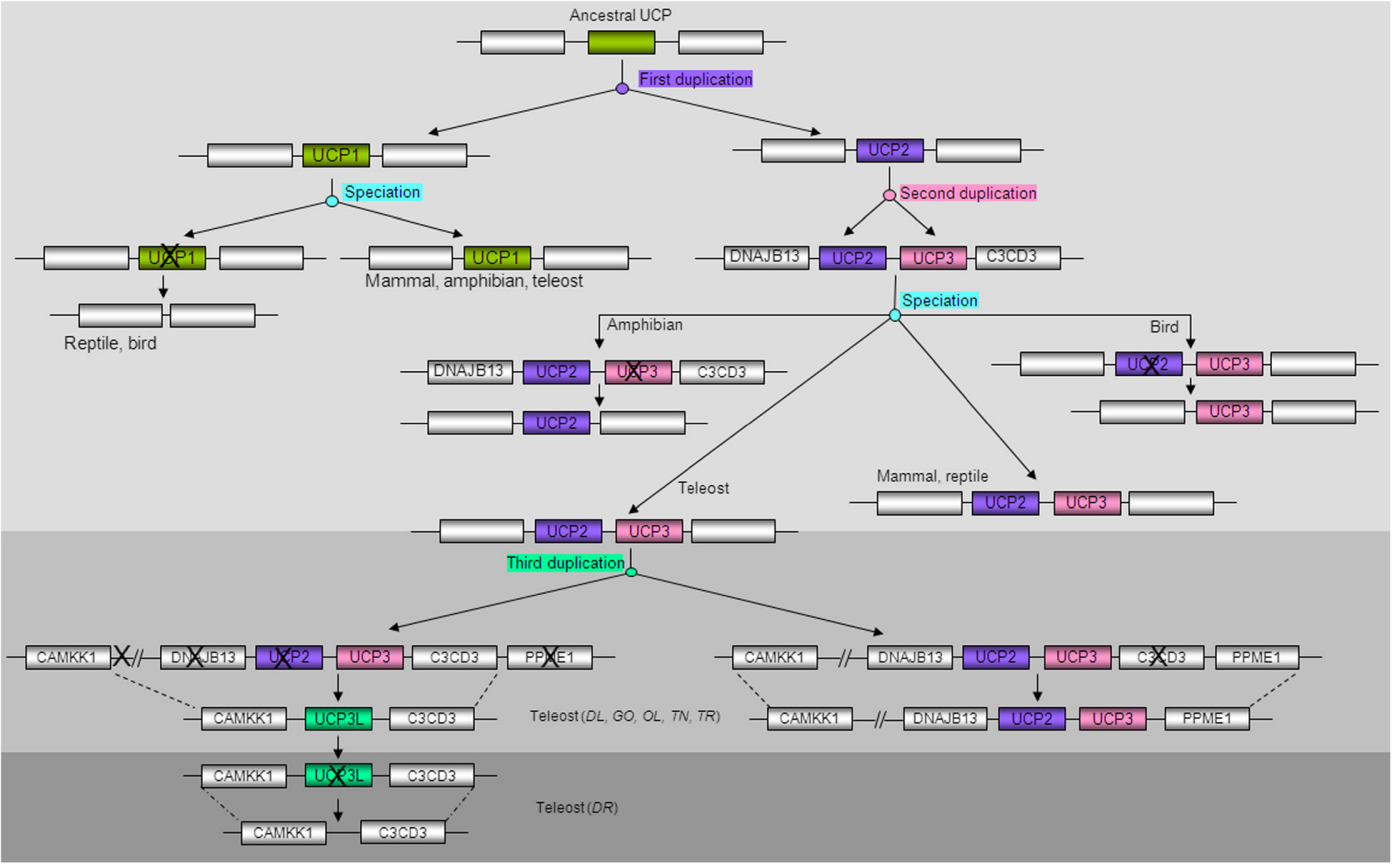
**Evolutionary scenario that produced vertebrate UCP genes.** The major evolutionary events (i.e. duplication and speciation) are highlighted in colors. The symbol (X) indicates genes that have been lost. Thus, UCP1 has been lost in reptile and bird lineages after the first duplication whereas UCP2 and UCP3 have respectively been lost in birds and amphibians after the second duplication. After the third duplication, C2CD3 was lost by the original fragment whereas all genes comprised between CAMKK1 and UCP3 including UCP2 were lost by the duplicated fragment. DL: *Dicentrarchus labrax*; GA: *Gasterosteus aculeatus*; OL: *Oryzias latipes*; TN: *Tetraodon nigroviridis*; TR: *Takifugu rubripes*; DR: *Danio rerio.*

On the other hand, sequence similarity did not enable the identification of the fourth teleost UCP gene in mammals, reptiles, birds and amphibians as well as in lampreys. With the analysis of conserved synteny of the genomic region harbouring the fourth UCP, we were unsuccessful in identifying its orthologue in other vertebrates. This indicates that our fourth UCP (UCP3L) might be a fish-specific gene and that the specific event which gives rise to that gene has occurred in teleost lineage and did not affect the other vertebrates. Interestingly, the downstream flanking gene of UCP3L was identical to that of UCP2/UCP3 and its upstream flanking gene (CAMKK1) was a locus located 3.7 Mb prior on the genomic sequence fragment harbouring UCP2/UCP3. Based on these observations and on the phylogenetic results, we suggest that the fourth teleost UCP resulted from a fish-specific duplication. Figure 7 indicates the evolutionary scenario that produced UCP3L in teleost fishes. The genomic fragment that harbors UCP2/UCP3 has been duplicated in teleost fishes. The C2CD3 locus was lost on the original fragment, possibly after the divergence of teleost and tetrapod ancestors, because of the presence of the latter in amphibians, reptiles and mammals. UCP3 as well as its flanking genes (CAMKK1 and C2CD3) were retained by the duplicated fragment whereas all genes between CAMKK1 and UCP3 including UCP2 were lost. The duplicated copy of UCP3 gene has diverged significantly from the original copy to become UCP3L whose retention suggests that it may have evolved indispensable functions in teleost organisms.

After divergence, UCP3L may have diverged in function by adopting part of the roles of the original copy or acquiring an entirely new function. Functional divergence can occur after gene duplication via site-specific rate shift (type-I functional divergence) or radical change in amino acid property, which is called type-II functional divergence. In this study, UCP3/UCP3L comparison showed θ_I_ value significantly greater than zero and negative θ_II_ value, suggesting that these two genes are functionally divergent from each other and that site-specific shift changes in evolutionary rates would have been the main force for such functional divergence. This conclusion is supported by the posterior probability analysis, which did not find any radical change between UCP3 and UCP3L clusters. Furthermore, the high d_N_/d_S_ ratio of UCP3L indicates that the protein is under few selective constraints and may have therefore evolved new functions. The overall d_N_/d_S_ ratio of UCP3L is about 1 and at least twice that of UCP3, which can be due either to a relaxation of negative or purifying selection or to an action of diversifying or positive selection. The REL analysis found no positively selected sites for UCP3L among teleost orthologues and the number of negatively selected sites is much lower than that of the other UCP members including UCP3. These results indicate that the higher d_N_/d_S_ ratios of UCP3L are due to a relaxation of purifying selection rather than positive selection. Such a relaxation of purifying selection can be attributed to a more recent origin of UCP3L, a finding which is consistent with the idea that recent duplicates tolerate more amino-acid substitutions than old duplicates [41]. It has been demonstrated that members of duplicated genes often evolve at different rates after duplication and that the copy (s) of the duplicated gene that evolve faster have higher d_N_/d_S_ ratios [42]. The results of the functional distance analysis showed relatively long functional branch length (bF = 0.35; Figure 6) for UCP3L, which implies that this duplicate gene may have lost a larger component of ancestral function after duplication. However, further analyses including tissue-specific expression and studies on protein function are needed to determine whether teleost UCP3 and UCP3L share communalities in function.

The comparison of the number of synonymous and nonsynonymous substitutions per site between UCP orthologues revealed that the products of UCP1-3 genes are highly conserved by purifying selection. This strong purifying selection against mutations that result in replacement of amino acids suggests that these UCP genes play crucial roles in teleost fishes. The examination of site-specific selection pressures by REL analysis identified only one site under moderate positive selection (BF ≥ 50) for UCP2 and UCP3 and our further analyses using more strict criteria (BF ≥ 100) did not support strong evidence for positive selection at these sites. Therefore, no valid conclusions could be drawn regarding the functional significance of these changes.

Analyses of vertebrate UCP evolution by Hughes and Criscuolo [27] and Saito et al. [43] suggest that UCP1-3 were acquired through two rounds of gene duplication. The first duplication produced UCP1 and the ancestral gene of UCP2/UCP3 whereas the second duplication produced UCP2 and UCP3. The results presented in this study confirm the previous conclusions that vertebrate UCP1-3 evolved from two successive duplications. The phylogenetic and syntenic analyses suggest that the first duplication (Figure 7) gave rise to UCP1 and UCP2/UCP3 ancestral gene took place prior to the divergence of vertebrates as evidenced by the presence of a homologue of UCP2 in early vertebrates (lampreys). The second round of duplication (Figure 7) that resulted in UCP2 and UCP3 occurred early in vertebrate evolution probably after the divergence of lampreys. Subsequent to these two duplications, UCP1 was lost from the avian and reptile lineages whereas UCP2 and UCP3 were lost from birds and amphibians, respectively (Figure 7). In addition to the two above mentioned duplications, a third round of duplication (Figure 7) occurred only in teleost fishes after they split from the tetrapod ancestors as evidenced by the absence of UCP3L in lampreys, amphibians, reptiles, birds and mammals. By taking a closer look at the chromosomal distribution of UCP genes in fish genomes, we found that UCP3L and UCP2/UCP3 are located on paralogous chromosomes in *T. nigroviridis*[44,45] and *O. latipes*[45]. These paralogous chromosomes arose from the whole genome duplication that occurred in ray-finned fish lineage around 350 million years ago [46-49]. Based on these observations, we speculate that UCP3L was generated in the ray-finned fish lineage as a result of a single event (fish-specific whole genome duplication) and not because of independent gene duplications.

The absence of UCP3L in zebrafish genome could thus be attributed either to the incompleteness of the genome assembly or to an independent loss of UCP3L in this species. To determine which of these phenomenon might be responsible for the UCP3L loss in zebrafish genome, we searched whether its flanking genes (CAMKK1 and C2CD3) are located on one or different contigs. We found that CAMKK1 and C2CD3 loci are located on contig CR450848.13, whose mapping to the sea bass genome assembly revealed higher synteny with chromosome 13 where UCP3L is located. Moreover, the re-annotation of the region harbouring CAMKK1 and C2CD3 loci did not reveal the presence of a UCP gene. Taken together, these observations suggest that UCP3L has been independently lost in zebrafish genome. Speculating about the reasons why UCP3L was lost only in zebrafish but not in the other teleost species is difficult without knowing the functional role of this gene in teleost fishes.

## Conclusions

By combining phylogenetic and detailed syntenic analyses, we unequivocally identified four UCP gene family members in teleost genomes, of which three (UCP1, UCP2 and UCP3) are shared by major vertebrate lineages. Phylogenetic results revealed that the gene duplication events that gave rise to these three genes occurred in the common ancestor of vertebrates, probably prior the divergence of lampreys. The fourth UCP (UCP3L) that has been identified only in fish, derived from teleost-specific third-round whole genome duplication that occurred in a common ancestor of teleost fishes. Functional divergence analysis among UCP family members indicated that substantial altered functional constraints have occurred on all UCP paralogues after gene duplication, which may have led to the evolution of new functions. These results suggest that UCP genes might be crucial for the adaptation of teleost to diverse ecological niches. The critical amino acid residues for functional divergence identified here provide new insights into the functional evolution and diversification of UCP genes, and since knowledge on UCP function is limited especially in fishes, they offer a starting point for further experimental validations.

## Methods

### Identification of scaffolds containing UCP genes and gene structure analysis

The methods applied for sequence data production of sea bass are described in full detail in [50,51]. To identify the scaffolds containing UCP genes, UCP1 protein sequence from stickleback, *Gasteroteus aculeatus* was blasted (TBLASTN, default parameters) against the sea bass draft genome assembly [Unpublished]. The resulting alignments were checked manually to identify the best hits considering score and identity values as well as a nearly complete alignment from start to end of the query. The matching scaffold fasta-files were converted to Game.xml databases and subsequently loaded into APOLLO Genome Curation Tool (V 1.9.6). *Ab initio* gene prediction was done with GENSCAN [52] to predict the genomic UCP gene structures. A homology-based prediction was conducted in parallel using UCP protein sequences from *G. aculeatus* using the SPALN software [53] together with APOLLO annotation curation tool. The stickleback proteins were used as informant sequences because its genome was identified to be the closest sequenced genome [49]. Since the SPALN output file is not compatible to APOLLO input format, the stickleback protein sequences were first splice-aligned to the genomic scaffold sequences with the SPALN software. Predicted exon coordinates of the SPALN output were then converted to a sim4 compatible format, which then was processed by APOLLO. The combination of both approaches provides more information that help to improve the accuracy of the final gene models. The modelling of genes in *D. labrax* was then performed manually by combining the results of GENSCAN and SPALN predictions and setting the start and stop codons. The gene models were tested and annotated (corresponding name) by BLASTP (default parameters) against nrprot database. The flanking genes of each UCP were predicted in each species by *ab initio* and homology-based prediction as described above for UCP genes. When an UCP orthologue could not be found in a given species, the region bounded by the flanking genes was extracted and re-annotated by *ab initio* and homology-based prediction to determine whether there was miss-annotation.

### Identification of sea bass UCP orthologues in other vertebrates

To identify orthologues in teleost fish species, the four UCP members identified in sea bass were blasted against zebrafish, medaka, takifugu, tetraodon and stickleback genomic sequences using the Ensembl genome browser server (Ensembl BLASTN, “distant homologies” settings). The upstream and downstream genes of each UCP member were identified in each species to determine whether they are conserved. The conservation of the flanking genes was then used to confirm that they are real orthologues of sea bass UCP members. Protein sequences of UCP gene family members of other vertebrates were also obtained from Ensembl genome browser or GenBank by BLASTP (Ensembl/NCBI default parameters) search. When a vertebrate orthologue could not be identified unambiguously, we searched for the flanking genes from fish to identify potential syntenic regions where UCP gene members would be located.

### Phylogenetic analysis

The predicted sea bass UCP amino acid sequences together with other teleost UCP genes as well as those identified in other vertebrates were aligned by MAFFT v6.864b [http://mafft.cbrc.jp/alignment/server] using default parameters. Amino acid sequence alignments were subsequently improved by removing the ambiguous positions using Gblocks [http://molevol.cmima.csic.es/castresana/Gblocks_server.html]. The JTT + G was selected as the best amino acid model on the final dataset comprised of total of 285 positions for teleost and 277 positions for all vertebrate including teleost using ProtTest v3.0 [http://darwin.uvigo.es/software/prottest3/prottest3.html]. The aligned amino acid data sets were used to construct the phylogenetic trees with maximum likelihood approach using PhyML v3.0 [http://www.atgc-montpellier.fr/phyml]. The teleost tree was rooted with invertebrate (*Ciona intestinalis*, *Ciona savignyi* and *Caenorhabditis elegans*) UCP sequences and the vertebrate tree with midpoint rooting as implemented in PhyML. The robustness of the tree topology was assessed by non parametric bootstrap analysis with 1,000 resampling replicates.

### Analysis of selection pressures

The selection pressure was estimated by comparing nonsynonymous (d_N_) and synonymous (d_S_) substitution rates (ω = d_N_/d_S_) for each individual UCP gene. A d_N_/d_S_ ratio of 1 is assumed as indicative of neutrality, d_N_/d_S_ > 1 is indicative of positive selection and d_N_/d_S_ < 1 is considered as a signature of purifying selection. Such analysis is mainly used to estimate the selective force acting on different alleles of a gene, but it can be also applied to duplicated genes with relatively low evolution rate, and which are not saturated in terms of synonymous substitutions. The d_N_/d_S_ ratios of all pair-wise comparisons of UCP genes were calculated using the yn00 program in PAML [54]. Additionally, we tested for selection among sites using Datamonkey online interface http://www.datamonkey.org which includes three methods [single likelihood ancestor counting (SLAC), fixed effects likelihood (FEL) and random effects likelihood (REL)] for detecting sites under selection. Among these methods, REL is the most powerful for detecting sites under selection particularly when dealing with small sample size of sequences or low sequence divergence. The REL method is an extension of codon-based selection analyses implemented in PAML. We have used REL method via the Datamonkey website instead of the models collected in the package PAML, because it allows both synonymous and nonsynonymous substitution rates to vary among sites. It has been demonstrated that the failure of modeling of d_N_ and d_S_ rate variation in a model can lead to misidentification of sites as positively selected [55] and the models collected in the package PAML do not allow modeling such variations. REL analysis calculates two Bayes factors (BF), one in the case of purifying or negative selection (d_N_ < d_S_) and another for positive selection (d_N_ > d_S_). A BF ≥ 50 is generally considered as an evidence for a moderate selection whereas a BF ≥ 100 indicates sites that have strong evidence positive selection. We considered sites to be under positive selection, if they met the initial criteria (BF ≥ 50) and further confirmed them with the more strict criteria (BF ≥ 100) for the detection of positive selection. The REL method was run on a neighbour joining phylogenetic tree after using the best substitution model (HKY85).

### Functional divergence

The coefficients of type-I and type-II functional divergence (θ_I_ and θ_II_) between any two UCP clusters were used to estimate the level of functional divergence among UCP paralogues (UCP1, UCP2, UCP3 and UCP3L). θ_I_ and θ_II_ were calculated for each amino acid position in the teleost UCP protein sequence alignment (see Additional file 4) using the method of Gu [56,57], as implemented in the DIVERGE v2.0 package. This method uses a maximum likelihood approach to assess significant changes in site-specific shift of evolutionary rate or site-specific shift of amino acid properties when comparing two clusters of duplicate genes. Type-I refers to functional divergence that occurred shortly after gene duplication as a result of site-specific changes in evolutionary rates between two clusters generated by gene duplication. In other words, it denotes amino acid residues that are conserved in one cluster but highly variable in the other, which implies that these residues have been subjected to different functional constraints. Type-II refers to amino acids that are highly conserved in both clusters but with very different chemical properties (i.e. positive versus negative charge). Large value of θ_I_ or θ_II_ (e.g. significantly greater than 0) supports the occurrence of functional divergence whereas θ_I_ or θ_II_ ≈ 0 indicates no functional divergence.

To determine which UCP cluster is functionally more divergent, we estimated the type-I functional distance between any two clusters, which is defined as dF = −ln(1 - θ_I_). Wang and Gu [58] have shown that under the assumption of independence between two clusters A and B, dF is additive i.e. dF (AB) = dF(A) + bF(B), where bF(A) and bF(B) are the functional branch length of clusters A and B, respectively. Large bF value for a duplicate cluster is indicative of altered functional constraints at many amino acid positions whereas bF ≈ 0 indicates that there was no significant shift in function from an ancestral function after gene duplication. The UCP functional branch lengths were also estimated in other vertebrates using their protein sequence alignment (see Additional file 4).

Furthermore, amino acid residues that might be crucial for type-I or type-II functional divergence among UCP paralogues were predicted using the site (k)-specific score Q_I_ (k) or Q_II_ (k). The site (k)-specific score corresponds to the posterior probability that site k is related to type-I or type II functional divergence.

## Authors’ contributions

MT, HK and RR conceived the study and participated in its design. MT performed the manuscript preparation. MJ and HK assisted in manuscript preparation and editing. HK performed sequencing and assembly of the derived sequence data. MT annotated the scaffolds and performed the phylogenetic and syntenic analyses. RR supervised and coordinated the project. All authors read and approved the final manuscript.

## Supplementary Material

Additional file 1 **Embl files of sea bass genomic UCP loci including flanking gene annotations.** These files contain the sequences of four UCP genes characterized in sea bass and their flanking genes. Click here for file

Additional file 2 **Pairwise comparisons of nonsynonymous and synonymous substitution rates of all teleost UCP genes.** Substitution rates were estimated using the yn00 program in PAML. Click here for file

Additional file 3 **Q**_**I**_**(k) or Q**_**II**_**(k) values for all combinations of pairwise comparisons of UCP clusters in teleost fishes.** Q_I_ (k) or Q_II_ (k) values highlighted in blue indicate sites that might be responsible for functional divergence. Click here for file

Additional file 4 **Multiple alignment of UCP protein sequences from six teleost species (A) and major vertebrate lineages including teleosts (B).** The amino acid alignments were conducted using MAFFT version 6 and the poor aligned regions were removed with Gblocks. The alignments were illustrated with GeneDoc. The most conserved amino acids are highlighted in black. Tetraodon: Te; Sea bass: Sb; Zebrafish: Ze; Stickleback: St; Medaka: Me; Takifugu; Fu; Xtrop: *Xenopus tropicalis*; A.liz: Anolis lizard; Hum: Huam; Mou: Mouse; Ele: Elephant; Lamp: Lamprey; Chick: Chicken. Click here for file
