# Supplementary material for: Genomic characterization of the European sea bass Dicentrarchus labrax reveals the presence of a novel uncoupling protein (UCP) gene family member in the teleost fish lineage
Source: BMC Evol Biol. 2012 May 11;12:62. doi: 10.1186/1471-2148-12-62 (PMC3428666; doi:10.1186/1471-2148-12-62)
Supplement: Additional file 4 — Multiple alignment of UCP protein sequences from six teleost species (A) and major vertebrate lineages including teleosts (B). The amino acid alignments were conducted using MAFFT version 6 and the poor aligned regions were removed with Gblocks. The alignments were illustrated with GeneDoc. The most conserved amino acids are highlighted in black. Tetraodon: Te; Sea bass: Sb; Zebrafish: Ze; Stickleback: St; Medaka: Me; Takifugu; Fu; Xtrop: Xenopus tropicalis; A.liz: Anolis lizard; Hum: Huam; Mou: Mouse; Ele: Elephant; Lamp: Lamprey; Chick: Chicken. [file 1471-2148-12-62-S4.doc]

A)

B)
